# Risk factors for cognitive dysfunction amongst patients with cardiovascular diseases

**DOI:** 10.3389/fpubh.2024.1385089

**Published:** 2024-09-13

**Authors:** Tunde Pal, Laszlo Barna Iantovics, Zoltan Preg, Eniko Nemes-Nagy, Kinga-Ilona Nyulas, Dragos-Florin Baba, Marta German-Sallo

**Affiliations:** ^1^Department of Internal Medicine V, George Emil Palade University of Medicine Pharmacy, Science, and Technology of Targu Mures, Targu Mures, Romania; ^2^Department of Cardiology, Emergency Institute for Cardiovascular Diseases and Transplantation of Targu Mures, Targu Mures, Romania; ^3^Department of Electrical Engineering and Information, George Emil Palade University of Medicine Pharmacy, Science, and Technology of Targu Mures, Targu Mures, Romania; ^4^Department of Family Medicine, George Emil Palade University of Medicine Pharmacy, Science, and Technology of Targu Mures, Targu Mures, Romania; ^5^Department of Cardiovascular Rehabilitation, County Emergency Clinical Hospital of Targu Mures, Targu Mures, Romania; ^6^Department of Chemistry and Medical Biochemistry, George Emil Palade University of Medicine Pharmacy, Science, and Technology of Targu Mures, Targu Mures, Romania; ^7^Department of Clinical Laboratory, County Emergency Clinical Hospital of Targu Mures, Targu Mures, Romania; ^8^PhD Student-Doctoral School, George Emil Palade University of Medicine Pharmacy, Science, and Technology of Targu Mures, Targu Mures, Romania; ^9^Department of Cell and Molecular Biology, George Emil Palade University of Medicine Pharmacy, Science, and Technology of Targu Mures, Targu Mures, Romania; ^10^Department of Internal Medicine III, George Emil Palade University of Medicine Pharmacy, Science, and Technology of Targu Mures, Targu Mures, Romania

**Keywords:** cognitive impairment, older adult, cardiovascular, risk factors, Montreal Cognitive Assessment, atrial fibrillation, stroke

## Abstract

**Background:**

The impact of cardiovascular diseases on cognition raises important research questions. The study aimed to investigate the relationship between demographic data, cardiovascular diseases, kidney disease and depressive symptoms on cognition.

**Methods:**

A cross-sectional study of patients with cardiovascular diseases was performed. The Montreal Cognitive Assessment (MoCA) was applied for cognitive evaluation. Based on MoCA three groups were defined: preserved cognition, mild, and advanced cognitive dysfunction (CD). Data were analyzed using Cronbach alpha (Cα) and McDonald’s ω (Mω) for internal consistency. The Chi-square test, Cramer’s V test, and correlation analyses were also applied.

**Results:**

Of 628 patients, 55.2% had mild CD, and the mean age was 67.95 (SD 9.53) years. Cα and Mω were 0.7, indicating good internal consistency. We found a moderate positive correlation between depression and the severity of CD (*r* = 0.25, *p* = 0.0001). A weak association between CD and female gender (*p* = 0.016), atrial fibrillation (*p* = 0.03), stroke (*p* = 0.009), and a moderate association for age group (*p* < 0.0001), education level (*p* < 0.0001), smoking (*p* < 0.0001), and renal dysfunction (*p* < 0.0001) was found. Age ≥ 70 years, eGFR 30–59 mL/min/1.73m^2^ significantly increased the likelihood for mild and advanced CD, while smoking and > 9 classes decreased it. Female gender, history of atrial fibrillation, and stroke significantly increased the likelihood of advanced CD.

**Conclusion:**

Mild CD was the most common in patients with cardiovascular diseases. Older age, lower education, being a non-smoker, and renal dysfunction were risk factors for both mild and advanced CD. Female gender, previous diagnosis of atrial fibrillation, and stroke are risk factors for advanced CD.

## 1 Introduction

Despite the enormous efforts of healthcare professionals, cardiovascular diseases (CVDs) and their complications have become a global burden on society and the economy due to the aging population (1, 2). Mild cognitive impairment is a transitional state between normal cognitive aging and dementia when activities of daily living are not limited. A considerable amount of literature demonstrated that CVDs and dementia share risk factors. According to the latest report of the 2020 Lancet Commission on dementia prevention, intervention and care, 12 potential risk factors may be responsible for the deterioration of cognitive function. Half represent classic cardiovascular risk factors (CVRFs): arterial hypertension, diabetes mellitus, smoking, high alcohol intake, obesity, and sedentarism (3). In Romania, dementia accounts for 1.43% of the population and is expected to rise to 2.56% by 2050 (4). This data refers to diagnosed dementia cases when the disease is usually advanced. Currently, there is no data about the prevalence of mild cognitive impairment in Romania.

Cognitive dysfunction (CD) and dementia mainly occur in the older adult population (4). However, in the Young Finns Study, CVRFs in childhood and adolescence were associated with lower cognitive function in midlife (5). Researchers along the heart-brain axis have studied the relationship between CD and cardiovascular pathologies (6). According to our knowledge, myocardial infarction, heart failure (HF) and atrial fibrillation (AF) are the most investigated pathologies lately. Ischemic heart disease, such as myocardial infarction or previous surgical revascularization for coronary heart disease and even high coronary calcium score were associated with an increased risk of CD development (7–9). AF is the most common arrhythmia with an increased risk for thromboembolism, often leading to ischemic stroke (10). Stroke plays an undisputed role in the deterioration of cognitive function (10). However, AF increases the risk of CD independently of stroke in older adults (11). Comorbid depressive symptoms are common in patients with CVDs and not only did they increase the short-and long-term risk for adverse cardiovascular events (12) but they also accelerated cognitive decline in a one-year follow-up (13).

Heart and brain health have much in common and this explains why this topic has received considerable interest nowadays. We hypothesized that individuals with different CVDs have worse cognitive performance and the impact on CD severity differs depending on the type of CVD. This study aimed to examine the prevalence of CD in the most common CVDs, such as atherosclerotic diseases, HF, AF and stroke. Furthermore, our objective was to evaluate the contribution and scale of the impact of demographic data, CVDs and two of the commonly co-occurring health conditions with CVDs, renal dysfunction (RD) and depression on cognitive deterioration.

## 2 Methods

### 2.1 Study population and design

This cross-sectional study enrolled participants from cardiology clinics in Mures County, Romania, between December 2016 and March 2022 (our study was stopped between March 2020 and January 2022 due to the coronavirus pandemic). Patients with CVDs admitted to the hospital were included based on past medical history records or actual medical data: ischemic heart disease, HF, AF, lower extremity peripheral artery disease and carotid artery disease. We considered ischemic heart disease based on the evidence of previous myocardial infarction (not recent), stable angina pectoris, coronary artery intervention (percutaneous or bypass surgery), significant artery stenosis (>50%), or occlusion demonstrated on coronarography. We included patients with significant peripheral artery stenosis (>50%) or occlusion demonstrated on angiography/Doppler ultrasound examination and prior percutaneous or surgical intervention for peripheral and carotid artery disease. In the case of AF, we included patients with established AF diagnosis and patients with AF on the admission electrocardiograms. For HF, previously established diagnoses, patients receiving guideline-recommended therapy, signs and symptoms of HF, and echocardiographic parameters were considered. HF patients with a left ventricle ejection fraction (LVEF) of less than 40% were considered as HF with reduced EF. A history of stroke was also reported. RD was determined based on the estimated glomerular filtration rate (eGFR) using the Modification of Diet in Renal Disease Study Group formula (MDRD, eGFR, mL/min/1.73m^2^). We divided patients into four groups: normal renal function: eGFR >90 mL/min/1.73m^2^, RD 2: eGFR 60–90 mL/min/1.73m^2^, RD 3: eGFR 30–59 mL/min/1.73m^2^, RD 4: eGFR <30 mL/min/1.73m^2^. Exclusion criteria were refusal to sign the written consent, the acute phase of any disease, recent (past 3 months) major cardio-or cerebrovascular events, patients on dialysis, or eGFR <15 mL/min/1.73m^2^. Other exclusion criteria were Alzheimer’s disease and other types of dementia, mental illness, or a disability that could hinder or affect cognitive tests, such as blindness, deafness, paresis, or paralysis of the dominant hand.

Anamnesis, sociodemographic data, physical examination, and laboratory tests were obtained from all patients. Risk factors for CVDs (arterial hypertension, diabetes, obesity, dyslipidemia, smoking) were recorded. Five age groups were established: Age I: 37–49 years, Age II: 50–59 years, Age III: 60–69 years, Age IV: 70–79 years, Age V:>80 years. We determined the educational level of 1–8 classes as low, 9–13 as intermediate, and > 13 as high.

The study protocol was approved by the Medical Science Committee of the George Emil Palade University of Medicine, Pharmacy, Science, and Technology of Targu Mures, the Emergency Institute for Cardiovascular Diseases and Transplantation of Targu Mures, and the County Emergency Clinical Hospital of Targu Mures, in accordance with the Declaration of Helsinki. All patients signed a written consent to participate in the study.

### 2.2 Applied questionnaires

The cognitive evaluation was carried out using the Montreal Cognitive Assessment (MoCA) questionnaire in the native language (Romanian, Hungarian) of the patients. Furthermore, we used the original ‘paper’ format. MoCA is a validated, efficient, and widely used screening test for the detection of mild cognitive impairment. It requires a standardized paper format and a pencil and usually takes 10 min to complete. The test has a maximum of 30 points and a cut-off value of 26 points (14). An extra point was added for an educational level of 12 classes or less. Based on the test results, three groups were determined: preserved cognition, scores between 26 and 30 points marked as MoCA 0, mild CD between 18 and 25 points (MoCA 1), and advanced CD <18 points (MoCA 2). In the advanced CD group, we combined moderate with severe CD, as the severe CD was not representative of the studied sample.

In addition to the cognitive examination, participants completed the shortened 13-item form of the Beck Depression Inventory (BDI) to detect depression as a confounding factor of cognitive performance.

### 2.3 Statistical analyses

#### 2.3.1 Internal consistency

We calculated Cronbach α (Cα) and McDonald’s ω (Mω) to verify the internal consistency of the 7 variables of the MoCA. We also calculated how the internal consistency is influenced by the individual variables (items) with the value of Cα for each variable if deleted.

#### 2.3.2 Descriptive statistics

Characteristics of participants were ordered by cognitive status. Welch’s test was applied for the comparison of means, as standard deviations were different, and the assumption was verified using the F-test. The difference between percentages was verified using the “N-1” Chi-squared test.

#### 2.3.3 Correlation analysis of cognitive dysfunction and depression

A positive linear correlation between cognitive dysfunction (MoCA) and depression (BDI) was verified. First, data normality was checked with the Lilliefors test (Lill), then we calculated the 95% CI of r and applied the ANOVA test.

#### 2.3.4 Independence analysis

Formulated hypotheses related to the independence of studied variables to CD were analyzed using the Chi-Square test. The Cramer’s V (Vcr) test was applied to measure the effect size for the obtained statistically significant hypotheses (Supplementary Table S1). It must be noticed that Cramer’s V produces relatively low measures, even for highly significant findings. Also, we have calculated the Odds Ratio (OR) and the 95% Confidence Interval (CI) for contingency tables by size 2×2. In the case of larger than 2×2 contingency tables, we analyzed the resulting 2 by 2 contingency tables obtained by decomposition and the OR and the 95% CI of OR were calculated.

#### 2.3.5 Hypotheses

Supplementary Table S2 presents the formulated hypothesis.

## 3 Results

### 3.1 Study sample characteristics

A total number of 628 patients participated in the study. Obtained Cα = 0.7 (0.697) and Mω = 0.7 (0.74) indicated a good internal scale consistency (Table 1). This result shows that the internal consistency cannot be increased significantly by removing any of the variables. Table 2 shows the baseline characteristics of the participants. The mean age of the cohort was 67.95 (SD 9.53) years, between 37 and 93 years with a median age of 69. Female gender was present in 52.2%. Approximately two-thirds of the participants were between 70 and 79 (37.7%) and 60 and 69 (32.8%) years. Most of the patients came from urban areas (56.8%) and had intermediate level of education (51.9%). Among the measured CVRFs, arterial hypertension was the most frequent (96.5%) followed by dyslipidemia in 68.6% and obesity in 52.7%.

**Table 1:** Cα and Mω value if variable/item dropped, with Cα = 0.697 and Mω = 0.74 for all included variables.

Variable (Cognitive domains)	If item dropped			
	Scale mean	Scale variance	Cα	Mω
Visuospatial/executive	18.99	14.03	0.62	0.69
Naming	19.63	19.85	0.69	0.74
Attention	17.82	13.37	0.62	0.69
Language	20.55	16.35	0.64	0.69
Abstraction	20.99	17.69	0.65	0.69
Delayed recall	20.04	14.86	0.71	0.74
Orientation	16.65	19.77	0.69	0.74

**Table 2:** Demographic data and clinical characteristics of study participants.

Characteristics	All *N* = 628	Preserved cognition (% from all)	Cognitive dysfunction (% from all)	*p*-value
Demographic data				
Gender *N* (%)	628 (100.0)	212 (33.8)	416 (66.2)	< 0.0001
Male *N* (%)	300 (47.8)	103 (34.4)	225 (65.6)	< 0.0001
Female *N* (%)	328 (52.2)	109 (33.3)	191 (66.7)	< 0.0001
Race				
White *N* (%)	628 (100.0)			
Age *N* (%)	628 (100.0)	212 (33.8)	416 (66.2)	< 0.0001
Mean age: year (SD)	67.95 (9.53)	64.01 (8.56)	69.96 (9.38)	< 0.0001*
Age I (<50 years) *N* (%)	19 (3.0)	9 (47.4)	10 (52.6)	0.75
Age II (50–59 years) *N* (%)	104 (16.6)	51 (49.1)	53 (50.9)	0.89
Age III (60–69 years) *N* (%)	206 (32.8)	93 (45.1)	113 (54.9)	0.047
Age IV (70–79 years) *N* (%)	237 (37.7)	54 (22.8)	183 (77.2)	< 0.0001
Age V (≥80 years) *N* (%)	62 (9.9)	5 (8.1)	57 (91.9)	< 0.0001
Provenience *N* (%)	525 (83.6)	190 (36.2)	335 (63.8)	< 0.0001
Urban *N* (%)	298 (56.8)	115 (38.6)	183 (61.4)	< 0.0001
Rural *N* (%)	227 (43.2)	75 (33.0)	152 (67.0)	< 0.0001
Educational level *N* (%)	507 (80.7)	181 (35.8)	326 (64.2)	< 0.0001
Low *N* (%)	149 (29.4)	21 (14.1)	128 (85.9)	< 0.0001
Intermediate *N* (%)	263 (51.9)	107 (40.6)	156 (59.4)	< 0.0001
High *N* (%)	95 (18.7)	53 (55.7)	42 (44.3)	0.12
Cardiovascular risk factors				
Arterial hypertension *N* (%)	628 (100.0)	212 (33.8)	416 (66.2)	< 0.0001
All cases *N* (%)	606 (96.5)	203 (33.5)	403 (66.5)	< 0.0001
Grade I. *N* (%)	22 (3.6)	5 (22.7)	17 (77.3)	0.0003
Grade II. *N* (%)	392 (64.7)	133 (34.0)	259 (66.0)	< 0.0001
Grade III. *N* (%)	192 (31.7)	65 (33.9)	127 (66.1)	< 0.0001
Smoking *N* (%)	308 (49.0)	117 (38.0)	191 (62.0)	< 0.0001
Active/Former smoker *N* (%)	142 (46.1)	67 (47.2)	75 (52.8)	0.35
Never smoker *N* (%)	166 (53.9)	50 (30.2)	116 (68.8)	< 0.0001
Dyslipidemia *N* (%)	576 (91.7)	193 (33.5)	383 (66.5)	< 0.0001
All types *N* (%)	395 (68.6)	128 (32.4)	267 (67.6)	< 0.0001
Hypercholesterolemia *N* (%)	205 (51.9)	69 (33.6)	136 (66.4)	< 0.0001
Hypertriglyceridemia *N* (%)	149 (37.7)	46 (30.8)	103 (69.2)	< 0.0001
Mixed *N* (%)	41 (10.4)	13 (31.7)	28 (68.3)	0.0010
Obesity *N* (%)	622 (99.0)	211 (34.0)	411 (66.0)	< 0.0001
All types *N* (%)	328 (52.7)	112 (34.1)	216 (65.9)	< 0.0001
Mean BMI: kg/m^2^ (SD)	30.76 (5.88)	31.35 (6.32)	30.46 (5.63)	0.009*
Grade I. *N* (%)	202 (61.6)	60 (29.7)	142 (70.3)	< 0.0001
Grade II. *N* (%)	80 (24.3)	30 (37.5)	50 (62.5)	0.0016
Grade III. *N* (%)	46 (14.1)	22 (47.8)	24 (52.2)	0.67
Diabetes *N* (%)	625 (99.5)	211 (33.8)	414 (66.2)	< 0.0001
*N* (%)	241 (38.5)	72 (29.8)	169 (70.2)	< 0.0001
Comorbidities				
Ischemic heart disease *N* (%)	180 (28.6)	65 (36.1)	115 (63.9)	< 0.0001
Heart failure *N* (%)	614 (97.8)	208 (33.8)	406 (66.2)	< 0.0001
Heart failure *N* (%)	334 (52.8)	95 (28.4)	226 (67.6)	< 0.0001
HFpEF *N* (%)	259 (77.6)	74 (28.5)	185 (71.5)	< 0.0001
HFrEF *N*⸙ (%)	75 (22.4)	26 (34.7)	49 (65.3)	0.0002
Mean EF % (SD)	61.03 (11.58)	60.88 (11.69)	61.11(1.54)	0.64*
Atrial fibrillation *N* (%)	178 (28.3)	48 (27.0)	130 (73.0)	< 0.0001
Carotid artery disease *N* (%)	52 (8.3)	13 (25.0)	39 (75.0)	< 0.0001
PAD *N* (%)	79 (12.6)	25 (31.7)	54 (68.3)	< 0.0001
Stroke *N* (%)	74 (11.8)	17 (23.0)	57 (77.0)	< 0.0001
Renal function *N* (%)	628 (100.0)	212 (33.8)	416 (66.2)	< 0.0001
All RD N (%)	392 (62.4)	114 (29.0)	278 (71.0)	< 0.0001
RD 2 N (%)	267 (68.2)	92 (34.5)	175 (65.5)	< 0.0001
RD 3 N (%)	117 (29.8)	20 (17.0)	97 (83.0)	< 0.0001
RD 4 N (%)	8 (2.0)	2 (25)	6 (75.0)	0.052
Depression *N* (%)	328 (53.9)	88 (26.9)	240 (73.1)	< 0.0001

For missing variables, the exact numbers were noted. Otherwise, the total number of cases was used. *Applied Welch’s test. Applied the “N-1” Chi-squared test for the rest. ^⸙^We considered reduced ejection fraction < 40%. BMI, body mass index; EF, ejection fraction; HFpEF, heart failure with preserved ejection fraction; HFrEF, heart failure with reduced ejection fraction; N, number; PAD, lower extremity peripheral artery disease; RD 2, eGFR 60–90 mL/min/1.73m^2^; RD 3, 30–59 mL/min/1.73m^2^; RD 4, eGFR < 30 mL/min/1.73m^2^; SD, standard deviation.

### 3.2 Results of cognitive assessment

**Figure 1:**
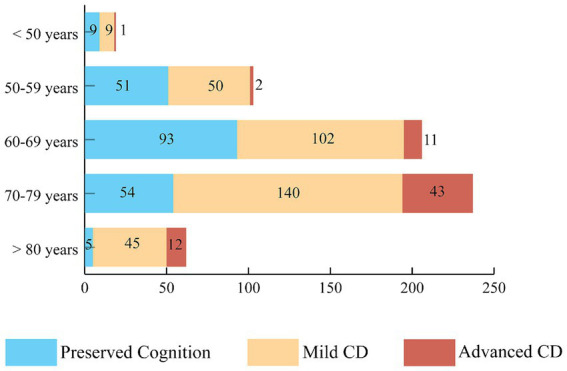
The prevalence of mild and advanced cognitive dysfunction. The distribution of CD severity based on MoCA test results in different age groups. More than half of the participants, 346 subjects (55.2%) had mild cognitive impairment and 69 patients (11.0%) had advanced CD. CD, cognitive dysfunction.

One patient was excluded from further analysis because of the lack of data about the severity of CD. We found cognitive impairment in 66.2% of patients, and overall, the mean MoCA score was 23.08 points, 20.84 points in the CD group and 27.45 points in the group with preserved cognition (Supplementary Table S3). Figure 1 demonstrates the distribution of the severity of CD in age groups.

#### 3.2.1 Cognitive dysfunction and depression

After eliminating patients with missing values, 607 cases were obtained for the correlation of depression and CD. We applied the Spearman correlation based on the Lilliefors test (Supplementary Table S4). A moderate positive correlation between depression and CD was revealed, *r* = 0.25, 95% CI: 0.17, 0.33, *p* = 0.0001.

#### 3.2.2 Association analysis result

The association analysis of all variables is summarized in Table 3. Female gender (*p* = 0.016), older age (*p* < 0.0001) and having a higher level of education (*p* < 0.0001) were associated with the severity of CD. The strength of the association was weak regarding female gender (Cramer’s V test *p* = 0.016) and moderate for older age group (Cramer’s V test *p* < 0.0001) and higher educational level (Cramer’s V test *p* < 0.0001). Active or former smoker status was associated with better cognitive function (*p* < 0.0001). Other CVRFs were not significantly related to CD. The severity of CD was found to be associated with AF (*p* = 0.03), stroke (*p* = 0.009), and RD (*p* < 0.0001). Cramer’s V test indicated a weak association between the severity of CD and AF (*p* = 0.033), respectively, between the severity of CD and the history of ischemic stroke (p = 0.009). In the case of RD, we found a moderate strength of association (*p* < 0.0001) (Table 4).

**Table 3:** Associations analysis results between variables and the severity of cognitive impairment.

Hypothesis	No valid cases	Ass passed	DF	p_ch_	χ^2^	Significant association
Gender	627	Yes	2	0.016	8.27	Yes
Age	627	Yes	8	< 0.0001	72.5	Yes
Education level	627	Yes	4	< 0.0001	67.22	Yes
Hypertension risk	605	Yes	4	0.08	8.47	No
Diabetes	624	Yes	4	0.4	4.05	No
Dyslipidemia	575	Yes	6	0.86	2.55	No
Smoking	308	Yes	2	< 0.0001	15.27	Yes
Obesity	621	Yes	2	0.15	3.87	No
IHD	627	Yes	6	0.17	9.03	No
HF	613	Yes	2	0.1	4.62	No
pEF	612	Yes	4	0.2	6.04	No
rEF	561	Yes	2	0.67	0.8	No
AF	627	Yes	2	0.03	6.85	Yes
PAD	627	Yes	2	0.67	0.81	No
CAD	627	Yes	2	0.19	3.38	No
Stroke	627	Yes	2	0.009	9.46	Yes
RD	627	Yes	4	< 0.0001	31.59	Yes

Column “No valid cases” includes the number of cases that do not contain missing values (cases with missing values excluded). AF, atrial fibrillation; CAD, carotid artery disease; DF, degree of freedom; EF, ejection fraction; HF, heart failure; IHD, ischemic heart disease; pEF, preserved ejection fraction; PAD, lower extremity peripheral artery diseases; rEF, reduced ejection fraction; RD, renal dysfunction.

**Table 4:** For significant associations, the obtained effect size and its interpreted strength.

Hypothesis	*p*-value of Cramer’s V test	Effect size	Strength of the association	PV (%)
Gender	0.016	0.115	Weak	0.0132 (1.32)
Age	< 0.0001	0.24	Moderate	0.0576 (5.76)
Education level	< 0.0001	0.232	Moderate	0.0538 (5.38)
Smoking	< 0.0001	0.222	Moderate	0.0493 (4.93)
AF	0.033	0.104	Weak	0.0108 (1.08)
Stroke	0.009	0.122	Weak	0.0149 (1.49)
RD	< 0.0001	0.219	Moderate	0.048 (4.8)

AF, atrial fibrillation; PV, proportion of variations; RD, renal dysfunction (the proportion of variation shared between two variables calculated based on the Phi coefficient).

Significant associations obtained were further analyzed with results presented in Table 5, reflecting the significant associations in preserved cognition (MoCA 0), mild cognitive impairment (MoCA 1), and advanced cognitive impairment (MoCA 2).

**Table 5:** The OR and 95% CI in MoCA 0, MoCA 1, and MoCA 2.

Hypothesis	Compared with	MoCA 0 and 1 OR (95% CI); *p*-value	MoCA 0 and 2 OR (95% CI); *p*-value	MoCA 1 and 2 OR (95% CI); *p*-value
Female	Male	–*	2.21 (1.25–3.93) *p* = 0.007	2.04 (1.18–3.53) *p* = 0.011
Age I (<50 years)	Age II (50–59 years)	–*	–*	–*
Age III (60–69 years)		–*	–*	–*
Age IV (70–79 years)		2.64 (1.60–4.36) *p* = 0.0001	40.61 (5.39–306.05) *p* = 0.0001	–*
Age V (≥80 years)		9.18 (3.37–25.03) *p* < 0.0001	122.40 (13.06–1147.20) *p* < 0.0001	–*
Education Intermediate	Low	0.41 (0.28–0.61) *p* < 0.0001	0.16 (0.08–0.30) *p* < 0.0001	0.37 (0.20–0.68) *p* = 0.0010
High		0.24 (0.14–0.40) *p* < 0.0001	0.04 (0.01–0.17) *p* < 0.0001	0.16 (0.04–0.70) *p* = 0.0140
Active or former smoker	Never smoker	0.57 (0.35–0.93) *p* = 0.0230	0.19 (0.08–0.48) *p* = 0.0230	0.34 (0.14–0.82) *p* = 0.0170
AF	No AF	–*	2.12 (1.18–3.80) *p* = 0.0120	–*
Stroke	No stroke	–*	3.13 (1.50–6.50) *p* = 0.0020	1.97 (1.04–3.73) *p* = 0.0380
RD2	No RD	–*	2.13 (1.06–4.30) *p* = 0.0350	–*
RD 3		2.77 (1.61–4.76) *p* = 0.0002	8.27 (3.73–18.37) *p* < 0.0001	2.99 (1.47–6.08) *p* = 0.0020

* = no significant association; AF, atrial fibrillation; CD, cognitive dysfunction; CI, confidence interval; MoCA 0, preserved cognition; MoCA 1, mild cognitive dysfunction; MoCA 2, advanced cognitive dysfunction; OR, odds ratio; RD, renal dysfunction; RD 2, eGFR 60–90 mL/min/1.73m^2^; RD 3, eGFR 30–59 mL/min/1.73m^2^.

Female gender increased the likelihood of advanced cognitive impairment (MoCA 2 OR: 2.21, 95% CI: 1.25–3.93), but not for mild cognitive impairment (MoCA 1). A higher age group increased the likelihood of mild CD (MoCA 1). The OR was 2.64 times greater for persons over 70 years (95% CI: 1.6–4.36) and 9.18 times greater for persons over 80 years (95% CI: 3.37–25.03) than those in their fifties. For advanced CD (MoCA 2), age presented the highest risk. The likelihood of odds increased to 40.61 (95% CI: 5.39–306.05) over 70 and to 122.4 (95% CI: 13.06–1147.2) over 80 years compared to patients between 50 and 59 years. More than 8 years of education decreased the likelihood of odds for mild and advanced CD (MoCA 1 and MoCA 2). The history of smoking reduced the likelihood of both mild and advanced CD (MoCA 1 OR: 0.57, 95% CI: 0.35–0.93; MoCA 2 OR: 0.19, 95% CI: 0.08–0.48). The presence of AF and stroke were associated with advanced CD (OR: 2.11, 95% CI: 1.18–3.8 and OR: 3.12, 95% CI: 1.5–6.5). RD 3 (30–59 mL/min/1.73m^2^) was a risk factor for both mild (OR: 2.76, 95% CI: 1.61–4.76) and advanced CD (OR: 8.27, 95% CI: 3.73–18.37). Significant associations were found between mild CD (MoCA 1) and advanced CD (MoCA 2). Female gender (OR: 2.04, 95% CI: 1.18–3.53, *p* = 0.011), previous stroke (OR: 1.97, 95% CI: 1.04–3.73, *p* = 0.038) and eGFR between 30 and 59 mL/min/1.73m^2^ (OR: 2.99, 95% CI: 1.47–6.08, *p* = 0.002) increased the likelihood of advanced CD (MoCA 2). Meanwhile, intermediate (OR: 0.37, 95% CI: 0.2–0.68, *p* = 0.001) and high level of education (OR: 0.16, 95% CI: 0.04–0.7, *p* = 0.014) and smoking (OR: 0.34, 95% CI: 0.14–0.83, *p* = 0.017) decreased it.

## 4 Discussion

**Figure 2:**
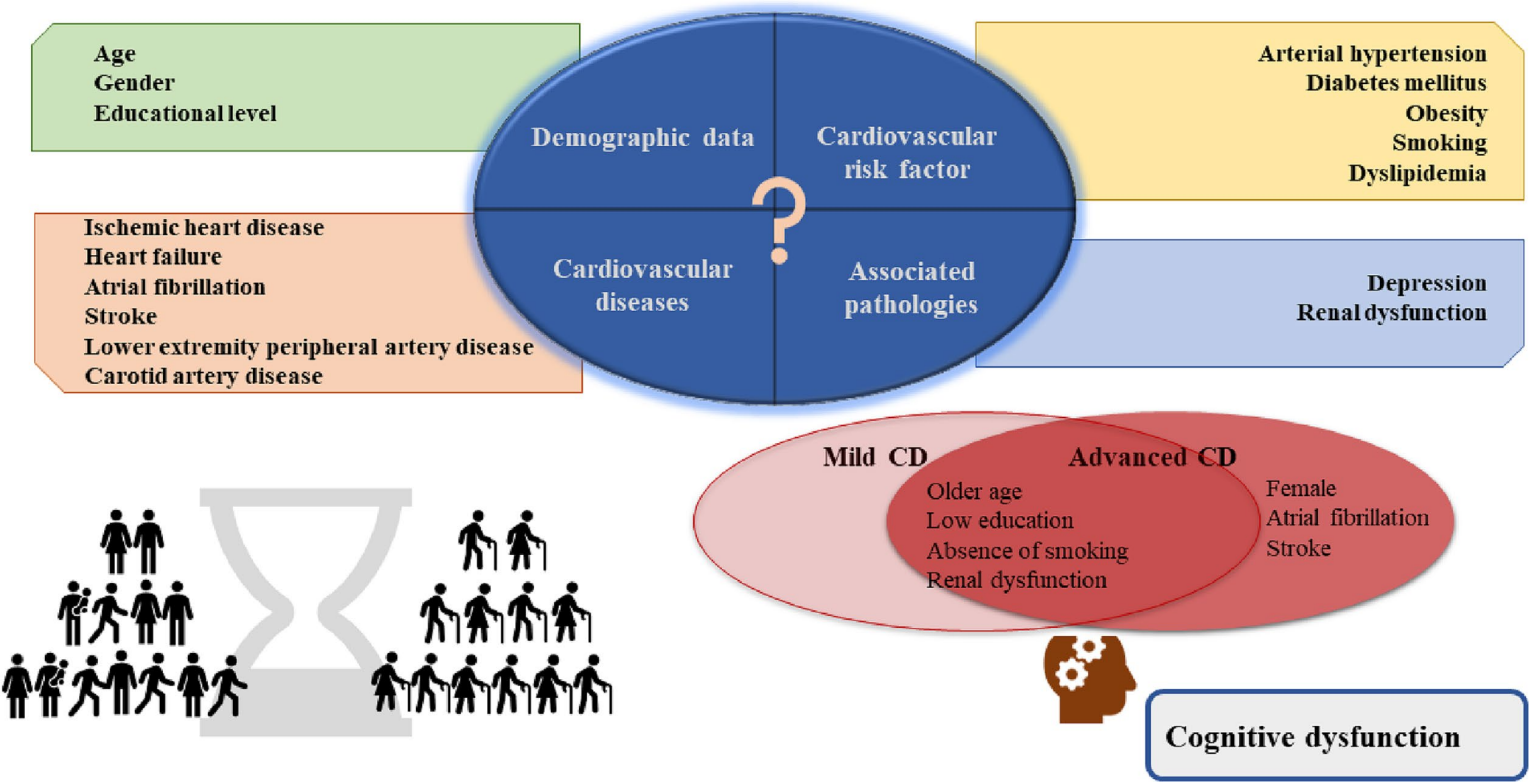
Central illustration. In the study sample, the risk factors for advanced cognitive dysfunction were female gender, previous diagnosis of AF and stroke; older age, lower education, absence of smoking, and renal dysfunction for both mild and advanced cognitive dysfunction. AF, atrial fibrillation.

According to the dementia forecast, the number of people living with CD is increasing due to the higher life expectancy and age is the main risk factor (2, 15). A considerable number of patients are not diagnosed (16). Since middle-aged and older adult patients commonly have multiple comorbidities, we thought it would be appropriate to investigate this patient profile. This study examined the prevalence of cognitive impairment in patients with CVDs in a Romanian sample. We found that cognitive impairment is high among patients with CVDs and the most common form is mild cognitive impairment. We investigated the relationship between cognitive impairment and gender, age, educational level, cardiovascular risk factors, the most common cardiovascular pathologies, and frequently associated conditions with CVDs, such as RD and depression. A significant association was found between gender, age group, level of education, smoking, AF, stroke, RD, depression and the severity of CD (Figure 2).

Researchers of the SHARE Database depicted a recent and authentic image of Europe from the CD point of view. Data was obtained from 16 European countries enrolling more than thirty thousand subjects aged 50 years or over. They found a decline in verbal fluency, perceived memory, and numeracy between 20.75 and 28.02% in the domains of the Mini-Mental State Examination test. In Hungary, another Eastern European country, a higher prevalence of impairment of perceived memory and verbal fluency (32.7 and 39.0%) was observed compared to global results (17). In the Romanian population, the only existing data is about dementia, whose prevalence was 279,607 individuals, representing 1.43% of the total population, according to the 2019 Dementia in Europe Yearbook (4). In our sample, the prevalence of CD was higher. We assessed CD in patients with established cardiovascular pathologies, contrary to Lourenco et al., where data referred to the general population (17). On the other hand, in a cross-sectional study conducted among community residents over 65 years, the overall incidence of cognitive impairment was 54.9% (18). These findings are closer to our results, not to mention that our research included patients with CVDs. Overall, the results highlighted the different prevalence of cognitive status between countries and may motivate other countries to obtain population data.

The present study’s findings are consistent with the main and established risk factor for cognitive decline (19). Older age (≥70 years) had a negative impact on cognition, both in mild and advanced CD. In addition, the female gender was a risk factor for advanced CD, which has already been confirmed in prior studies (20, 21). Furthermore, in line with other studies, we found that higher education is a protective factor for cognitive function. Longitudinal studies suggested that nine and 10 classes could be determinative in preserving cognitive abilities (22, 23). Our results confirmed that 9 years of education could be the threshold.

Despite expectations based on previous research (6, 17, 24), our study did not demonstrate a significant association between CVRFs and cognitive impairment, except for smoking. Unlike some previous reports, our correctly applied statistically grounded result on smoking in the studied cohort of patients indicated a different finding. In the present paper, smoking appeared to be a protective factor for mild and advanced CD. Smoking cessation is one of the potentially modifiable risk factors to prevent all types of dementia (3). Moreover, a meta-analysis revealed that active and former smokers had a significantly increased risk for all-cause dementia, Alzheimer’s disease, and vascular dementia compared to non-smokers. This association was weaker in former smokers than in current smokers (25). This may further favor smoking cessation. However, a limited body of evidence showed a positive effect of smoking on various cognitive domains, such as attention, short-, and long-term memory (26). It was suggested that the nicotine compound of the cigarette could be responsible for this improvement (27). Moreover, the administration of lower-dose nicotine gum in young non-smokers showed improvement in cognitive performance (28). Taking into consideration the massive scientific data about the harmful effects of smoking (29), our result that smoking might have a positive effect on cognition in this patient sample highlights the gaps in evidence and implies future investigation of the research team. From blood-derived CVRFs, such as glucose, lipid profile, homocysteine, high sensitivity C-reactive protein, only increased high-density lipoprotein cholesterol level was associated with better cognitive function after 2 years in individuals with normal baseline cognitive function (30). Similar results were found in 1324 community residents, where the incidence of CD was significantly higher in individuals with arterial hypertension and low level of low-density lipoprotein cholesterol, but not with other CVFRs (18). In addition, heart diseases and risk factors sometimes overlap. Grouping cardiovascular pathologies in cognitive studies is more common in the literature than assessing a single disease. In a study with self-reported CVDs such as “heart attack, coronary heart disease, angina, congestive heart failure or other heart pathologies” there was no increase in the risk of CD compared to patients without CVDs for 8 years of follow-up (31). A Mexican study analyzing 1807 subjects concluded that CVDs in mid-life, but not late-life disease, increased the likelihood of mild cognitive impairment by 1.7 times. In this study, cumulative CVDs, such as arterial hypertension, diabetes mellitus, heart disease, and stroke, were considered. Further evaluation of one single disease from those mentioned above demonstrated that arterial hypertension and diabetes mellitus in mid-life and late-life heart disease increased the risk of mild cognitive impairment (32). It must be mentioned that this longitudinal study also applied self-reported CVDs. The main practical problem that concerns us is implementing these results in a middle-aged or older adult patient with more than one pathology. These results may suggest that the relationship between CVDs and cognitive decline should be assessed separately for pathologies. In contrast to other colleagues, we included patients with documented pathologies, as self-reported or anamnestic health conditions may mask biases.

A large body of evidence supports that ischemic heart disease, AF, and stroke are risk factors for vascular dementia (19). Previous diagnostics of AF and ischemic stroke increased the risk for advanced cognitive decline in our study, but not for mild CD with a weak effect size. Clinical data showed that not only did AF represent a risk factor for CD (19) but a longer history of AF was suggested to lead to faster deterioration of cognitive performance (33). Advanced CD, such as dementia, may raise concerns, as deterioration of cognitive domains interferes with activities of daily living (3). Our findings suggest that AF and a history of stroke similarly impact cognitive function. Considering that some of the risk factors for CD from our results are non-modifiable, such as age, gender, and educational level, the presence of AF could be a modifiable risk factor in some instances. However, data are insufficient to support rhythm or frequency control in AF patients to prevent CD (34). Anticoagulation therapy could offer a protective effect against CD in AF patients, although further research is needed to confirm this hypothesis (34, 35). Stroke-related decline in cognitive function is an acknowledged complication of stroke (36, 37). Unfortunately, few patients can expect to recover from it (38). Even though contrary results have been reported too, in a study with 7 years of follow-up the incidence of CD in patients with and without stroke was not significantly different (39). The outcome of cognitive performance after stroke is uncertain, but cognitive reserve may represent a protective factor (40).

Insufficient data is available in the case of low cardiac output conditions (19). Approximately half of the participants had a history of heart failure, which was in accordance with other studies (41). Even though the prevalence of CD was high in HF patients in our study, no association between HF and the severity of CD was found, regardless of the HF type and level of EF. Prior research has found an increased risk for CD in patients diagnosed with HF either with reduced or preserved EF (HFrEF, HFpEF) (42) and in any reduction of LVEF (43, 44). Even the NYHA class of ≥II was associated with the decline of cognitive function in patients with HFrEF (45). Nevertheless, it is needed to mention that 77% of HF patients in the present study were documented with preserved ejection fraction, the patients were relatively well compensated and there was no need for high doses of loop diuretics and inotropic/vasopressor usage.

We found a considerable proportion of patients who had comorbid conditions such as RD and depressive symptoms. A systematic review and meta-analysis revealed that the prevalence of depression was 32% in patients with CD, with a higher proportion in clinical studies compared to community studies (46). Depressive symptoms were positively correlated with CD. RD was associated with the severity of CD and increased the risk of cognitive decline with moderate association strength. An eGFR between 30 and 59 mL/min/1.73m^2^ increased the likelihood for mild and advanced CD, while eGFR 60–90 mL/min/1.73m^2^ for advanced CD. In the “Reasons for Geographical and Racial Differences in Stroke” (REGARDS) study, eGFR<60 mL/min/1.73m^2^ was associated with CD (47) and it was also demonstrated in HFrEF patients measured by the Mini Mental State Examination test (45).

Although most of the research in the field of cognitive impairment among patients with CVDs was performed on older adult individuals, midlife CVDs also hide a risk for cognitive decline in younger patients. A longitudinal study over 30 years has found that cognitive dysfunction was associated with premature CVDs (the term of premature denoted patients <60 years) and with a higher percentage of white matter hyperintensities on brain magnetic resonance images (48). These findings emphasize whether the key factor in maintaining cognitive function is the prevention of CVDs. This research question was aimed at the FINGER trial (Finnish Geriatric Intervention Study to Prevent Cognitive Impairment and Disability). The authors revealed that intensive control and management of risk factor as well as specific recommendations and interventions related to cognitive training and healthy lifestyle, such as advice on diet, physical activity, and weight loss, contributed significantly to the reduction in cognitive decline in older patients from the general population at risk for dementia (49).

While other studies searched for an association between risk factors, diseases, and CD, we investigated whether there is a difference between these conditions and the severity of CD. Few studies assessed the strength of the association between demographic data, CVDs, and related pathologies and cognitive decline. The novelty of this study lies in more aspects. Firstly, we revealed the prevalence of CD in a high-risk sample and also found that the most common form of CD was mild CD. Secondly, we demonstrated that some of the investigated pathologies hide a greater risk for advanced CD, such as atrial fibrillation and stroke in comparison with mild CD. Furthermore, we investigated CVDs separately against global CVDs.

An obvious limitation of our study was the observational design and the absence of a control group from the general population or primary health services. Our study may overestimate the prevalence of CD in the general population since we included patients hospitalized for specific reasons, sometimes with multiple CVDs. In addition, we utilized a cognitive test that assesses various cognitive domains, but instead we focused on global cognition. Furthermore, given that we conducted a cross-sectional study, cognitive performance was measured once. All authors agree that longitudinal follow-up is needed for more robust findings and conclusions.

## 5 Conclusion

The findings of the present manuscript show that in patients with CVDs mild cognitive impairment was more common than advanced (moderate or severe) CD. AF and stroke were associated with the severity of cognitive decline with a weak association of strengths. Moreover, AF, stroke and female gender increased the probability of advanced CD but did not influence mild CD. The likelihood for mild and advanced CD was increased by advanced age, lower level of education, renal dysfunction, and being a non-smoker. The clinical implications of these findings remain subject to future investigations.

## Data Availability

The raw data supporting the conclusions of this article will be made available by the authors, without undue reservation.
