# Supplementary material for: Risk factors for cognitive dysfunction amongst patients with cardiovascular diseases
Source: Front Public Health. 2024 Sep 13;12:1385089. doi: 10.3389/fpubh.2024.1385089 (PMC11427290; doi:10.3389/fpubh.2024.1385089)
Supplement: Supplementary file 1 [file Table_1.DOCX]

Supplementary Material

# Supplementary Tables

## Supplementary Table S1

Interpretation of Cramer's V Values

| V_cr_ | Strength of association |
| --- | --- |
| [0.8,1] | Very strong |
| [0.6,0.8) | Strong |
| [0.4,0.6) | Relatively strong |
| [0.2,0.4) | Moderate |
| [0.1,0.2) | Weak |
| [0,0.1) | Negligible |

## Supplementary Table S2

The formulated Hypothesis

| Hypothesis nr. | Formulated hypothesis |
| --- | --- |
| Statistical modeling: Correlation analysis | |
| HA | It is a positive correlation between depression and CD severity. |
| Statistical modeling: Association analysis | |
| H1 | There is an association between gender and CD severity. |
| H2 | There is an association between age and CD severity. |
| H3 | There is an association between educational level and CD severity. |
| H4 | There is an association between hypertension risk and CD severity. |
| H5 | There is an association between diabetes and CD severity. |
| H6 | There is an association between dyslipidemia and CD severity. |
| H7 | There is an association between smoking and CD severity. |
| H8 | There is an association between obesity and CD severity. |
| H9 | There is an association between ischemic heart disease and CD severity. |
| H10 | There is an association between heart failure and CD severity. |
| H11 | There is an association between atrial fibrillation and CD severity. |
| H12 | There is an association between peripheral artery disease and CD severity. |
| H13 | There is an association between carotid artery disease and CD severity. |
| H14 | There is an association between stroke and CD severity. |
| H15 | There is an association between renal dysfunction and CD severity. |
| H16 | There is an association between heart failure with preserved and reduced ejection fraction and CD severity. |
| H18 | There is an association between ejection fraction and CD severity. |

H, hypothesis; nr, number; CD, cognitive dysfunction.

## Supplementary Table S3

Descriptive Statistics of MoCA Test Results

| MoCA results | Number | Mean (points) | Minimum (points) | Maximum (points) | Range (points) | Median(points) | SD (points) | RSD (points) |
| --- | --- | --- | --- | --- | --- | --- | --- | --- |
| MoCA 0 | 212 | 27.45 | 26 | 30 | 4 | 27 | 1.27 | 0.046 |
| MoCA 1 | 415 | 20.84 | 7 | 25 | 18 | 22 | 3.91 | 0.187 |

MoCA, Montreal Cognitive Assessment; MoCA 0 ≥26 points, preserved cognition; MoCA 1 <26 points, cognitive dysfunction; SD, standard deviation; RSD, relative standard deviation.

## Supplementary Table S4

Lilliefors Test Results for Variables MoCA and BDI

| Variable | Lilliefors test | | |
| --- | --- | --- | --- |
|  | Statistic | df | p-value |
| MoCA | 0.13 | 607 | <0.00001 |
| BDI | 0.14 | 607 | <0.00001 |

BDI, Beck Depression Inventory; df, degree of freedom; MoCA, Montreal Cognitive Assessment.
